# Delay

**Published:** 1867-07

**Authors:** 


### *Delay.*

The issue of THE JOURNAL for July has been delayed for a few days, in order to send out with it the Annual Announcement and Catalogue of Rush Medical College. It will be seen that the school is in a condition of high prosperity, and its prospects for the future more flattering than ever before during its history.

